# Determination of Isothiocyanate-Protein Conjugates in a Vegetable-Enriched Bread

**DOI:** 10.3390/foods10061300

**Published:** 2021-06-05

**Authors:** Mareike Krell, Lina Cvancar, Michael Poloczek, Franziska S. Hanschen, Sascha Rohn

**Affiliations:** 1 Institute of Food Chemistry, Hamburg School of Food Science, University of Hamburg, Grindelallee 117, 20146 Hamburg, Germany; mareike.krell@chemie.uni-hamburg.de (M.K.); Lina.Cvancar@gmx.de (L.C.); m-poloczek@gmx.de (M.P.); 2 Leibniz Institute of Vegetable and Ornamental Crops (IGZ) e.V., Theodor-Echtermeyer-Weg 1, 14979 Großbeeren, Germany; hanschen@igzev.de; 3 Institute of Food Technology and Food Chemistry, Technische Universität Berlin, Gustav-Meyer-Allee 25, 13355 Berlin, Germany; 4 Institute for Food and Environmental Research (ILU) e.V., Papendorfer Weg 3, 14806 Bad Belzig, Germany

**Keywords:** glucosinolates, benzyl isothiocyanate, protein conjugates, functional foods, nasturtium, garden cress, thiourea

## Abstract

Vegetables of the plant order Brassicales are believed to have health-promoting properties, as they provide high contents of glucosinolates (GLS) and deriving from these, enzymatically and heat-induced breakdown products, such as isothiocyanates (ITC). Besides their positive physiological effects, ITC are electrophilic and can undergo reactions with food components such as proteins. Following the trend of improving traditional food products with GLS-rich ingredients, interactions of ITC with proteins can diminish the properties of both components—protein’s value and functionality as well as ITC’s bioactivity. In vegetable-enriched bread, where cresses (*Lepidium sativum* L. or *Tropaeolum majus* L.) are added to the initial dough, together with benzyl cyanide, benzyl isothiocyanate (BITC) is formed during the baking process. The aim of the present study was to investigate the possible migration behavior of the GLS breakdown products and the formation of ITC-wheat protein conjugates. After the baking process, the breads’ proteins were enzymatically hydrolyzed, and the ITC-amino acid conjugates analyzed using a LC-ESI-MS/MS methodology. In all samples, BITC-protein conjugates were detected as thiourea derivatives, while formation of dithiocarbamates could not be detected. The study showed that GLS and their breakdown products such as ITC migrate into the surrounding food matrix and undergo reactions with proteins, which could in turn lead to modified protein properties and reduce the bioavailability of ITC and lysine.

## 1. Introduction

Plants possess a wide variation of bioactive compounds derived from plant secondary metabolism. When consumed these can provide pharmacological properties and beneficial effects on human health [1,2]. Consequently, the *World Health Organisation* (WHO) recommends an intake of at least 400 g of fruits and vegetables per day, which is still not being reached sufficiently [3,4]. In order to further increase the supply of vegetables with their health-promoting secondary plant metabolites (SPM), different pasta and bread recipes have already been adapted, applying raw materials rich in SPM [5,6,7]. Bread seems to be a particularly suitable product for a fortification with SPM, as it is consumed quite frequently in Western countries, regardless of age and gender [8].

When vegetables of the plant order Brassicales are used as ingredients in foods, glucosinolates (GLS) are in many cases the dominating SPM [9]. Besides antibacterial and anti-inflammatory effects, some studies even suggest a reduced risk of suffering from certain types of cancer, when vegetables containing GLS are regularly consumed [10,11]. However, the health-beneficial effects seem to be not directly associated with the native GLS, but rather linked to their breakdown products, especially isothiocyanates (ITC) [12]. In addition to ITC, nitriles, thiocyanates, and epithionitriles can be formed after the damage of the plant tissue, as occurring during chewing or cutting. During food processing nitriles are formed via thermally-induced degradation [13,14]. The tissue damage causes the enzyme myrosinase to come into contact with the GLS and hydrolyze them into unstable aglycone intermediates. These are then further converted by a Lossen-like rearrangement or specific proteins into the different breakdown products [15,16]. The profile of the breakdown products formed is dependent on the pH value, the presence of modifier proteins, and the initial composition of the GLS [17].

Due to their electrophilic nature, ITC can react with nucleophiles such as the amino or thiol groups of amino acids, peptides, or proteins. The reaction of ITC with the side chains leads to the formation of thiourea and dithiocarbamate derivatives, respectively, presenting different stabilities and subsequent reaction pathways [18,19,20]. Conjugates between ITC and different food proteins such as myoglobin, egg white proteins, and whey proteins have already been studied. It was found that the ITC-protein conjugation does not only lead to altered physicochemical properties, but also to a structural change of the proteins [18,21,22]. In food, ITC-protein conjugates were already identified when garden cress (*Lepidium sativum*) was mixed with curd (as in the typical German spread ‘Kräuterquark’) with the result that 28% of the original GLS were conjugated to lysine and cysteine [23].

In a recent study, bread was enriched with pak choi (*Brassica rapa* subsp. *chinensis*) and kale (*Brassica oleracea* var. *sabellica*) for increasing the intake of GLS and their breakdown products [24]. During breadmaking, the ingredients undergo a heating process, reaching temperatures around 100 °C. Consequently, mainly nitriles rather than ITC were detected in those experiments, as higher temperatures conditions preferably lead to the formation of nitriles, which have a higher thermostability [24,25]. The desired health-promoting ITC were only found in bread enriched with pak choi (*Brassica rapa* subsp. *chinensis*) microgreens in a concentration below 0.005 µmol/g fresh weight. Further reasons for the low ITC concentration could have been based on the chemical structure of the GLS, the water content of the bread, and the plant tissue being only marginally destroyed [24]. The behavior of ITC in a processed food with regard to interactions with nucleophilic amino acid side chains was not yet investigated in detail. However, reactions between ITC from the added vegetables and proteins in a more highly processed product such as bread, still need characterization, as fate of the GLS and ITC during breadmaking is not yet completely understood. Such interactions can affect the physiological as well as the technofunctional properties of both components.

It is hypothesized that the thermally-induced breakdown products of GLS can migrate into the bread crumb and form ITC-wheat protein conjugates to a certain extent, away from their place of formation. Consequently, the aim of this study was to investigate the formation of ITC, their migration behavior in a model bread matrix, and the possible conjugation with wheat proteins. Based on a recent experiment, wheat dough was used, mixed with fresh garden cress (*Lepidium sativum* L.) or freeze-dried nasturtium leaf-powder (*Tropaeolum majus* L.) [7]. Microgreens and homogenized material from the different cress genera were used for studying the factors that can influence the formation of ITC conjugates such as different BITC sources and the form of plant-material addition.

## 2. Materials and Methods

### 2.1. Chemicals and Materials

Boric acid solution (4%), hydrochloric acid (0.1 M), hydrochloric acid (32%), formic acid (FA; 98%), sodium hydroxide (100%), trichloroacetic acid (≥99%), Kjeldahl tablets, sulfuric acid (96–98%, p.a.), methanol (LC-MS grade), water (LC-MS grade), methylene blue, methyl red and methylene chloride (GC Ultra Grade, solvent) were purchased from Carl Roth GmbH & Co. KG (Karlsruhe, Germany). Pronase E (from *Streptomyces griseus*), pepsin (from porcine gastric mucosa), pancreatin (from porcine pancreas), β-glucuronidase (from *Helix pomatia*), DEAE Sephadex^®^ A-25, 4-hydroxybenzyl glucosinolate potassium salt, 2-propenyl glucosinolate potassium salt, benzyl cyanide (≥98%), benzonitrile (≥99.9%), and benzyl isothiocyanate (≥98.5%) were obtained from Merck KGaA (Darmstadt, Germany). 1*H*-Imidazole was purchased from AppliChem GmbH (Darmstadt, Germany), boric acid (powder, pure) from Honeywell International Inc. (Seelze, Germany) and 4-(methylsulfinyl)butyl glucosinolate from PhytoLab GmbH & Co. KG (Vestenbergsgreuth, Germany). Sodium sulfate anhydrous (≥99%) was obtained from VWR International GmbH (Darmstadt, Germany). C18ec solid phase extraction cartridges (3 mL, 200 mg) were obtained from Machery-Nagel GmbH & Co. KG (Düren, Germany). The BITC-lysine and BITC-cysteine standards used were synthesized by Kühn et al. [23].

### 2.2. Plant Material

Garden cress seeds (*Lepidium sativum* L.) were purchased from Dürr Samen (Reutlingen, Germany). The seeds were cultivated for seven days at room temperature on cotton wool with natural light on a windowsill and sprayed with water every day. The sprouts were harvested and divided into two parts. One part was used for the analysis of the GLS content of the non-processed material, the other part was directly applied as ingredient in the bread dough.

Freeze-dried nasturtium (*Tropaeolum majus* L.) leaves were obtained from the Leibniz Institute of Vegetable and Ornamental Crops (IGZ) e.V. (Großbeeren, Germany). The cultivation was done according to standard procedures for *Brassica* vegetables, including fertilization, irrigation, and plant protection. After 10 weeks, leaves were harvested and immediately frozen at −50 °C, freeze-dried, and milled to a fine powder [26]. A part of the homogenized material was analysed for the GLS content and the other part applied into the bread dough.

### 2.3. Bread Baking and Material for Analysis

Three bread types with different cress applications and percentage addition relative to the dough weight were prepared: freeze-dried, powdered nasturtium leaves (*Tropaeolum majus* L., 4%) mixed homogenously into the dough (application No. I); parts of fresh garden cress microgreens (*Lepidium sativum* L., 1.5%) mixed into the dough (application No. II), and freeze-dried nasturtium leaves (*Tropaeolum majus* L., 1.5%) placed centrally in the dough (application No. III).

**Figure 1:**
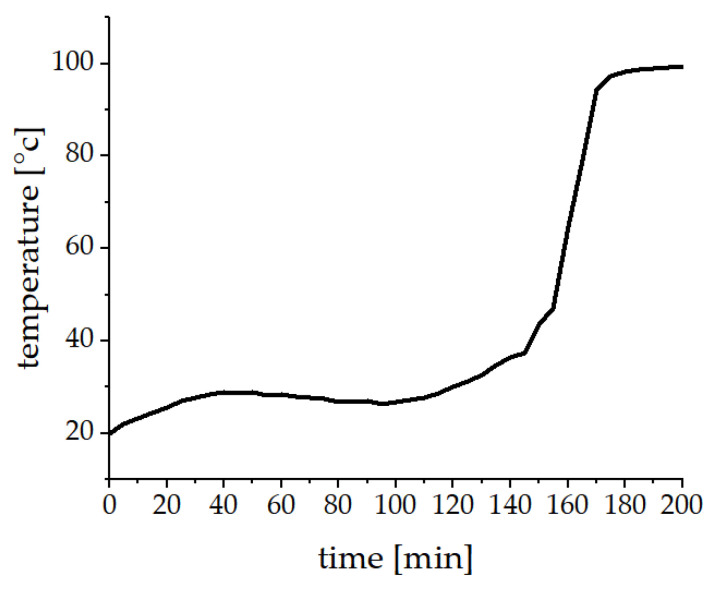
Temperature profile of the rise and baking in the bread maker (UNOLD Backmeister^®^ extra Modell 65811, UNOLD AG, Hockenheim, Germany).

The different bread types were prepared in triplicate. The basis was a wheat dough with 65% wheat flour (Type 450, EDEKA Zentrale AG & Co. KG, Hamburg, Germany), 33% water, 1% salt, 0.5% sugar, and 1% yeast (EDEKA Zentrale AG & Co. KG). The dough was prepared by adding the water to the dry ingredients and kneaded by hand for 5 min. The dough was divided, and the plain dough was used as control. The second part of the dough was mixed with freeze-dried powdered nasturtium leaves (application No. I) or respectively with parts of fresh garden cress microgreens (application No. II). The resulting levels of benzyl glucosinolate (BG) were around 50 µmol/100 g bread dough for application No. I and 20 µmol/100 g bread dough for application No. II. For the rise and the baking, a commercially available breadmaking device (UNOLD Backmeister^®^ extra Modell 65811, UNOLD AG, Hockenheim, Germany) was used. The program sequence is listed in Table 1 and the temperature profile is given in Figure 1. After cooling down the whole bread, the crust was removed, and the crumb was frozen at −80 °C. For the analysis of GLS breakdown products the material was grounded using liquid nitrogen to prevent thawing and stored frozen at −80 °C. To investigate ITC-protein conjugates lyophilized powder was used, also stored at −80 °C.

**Figure 2:**
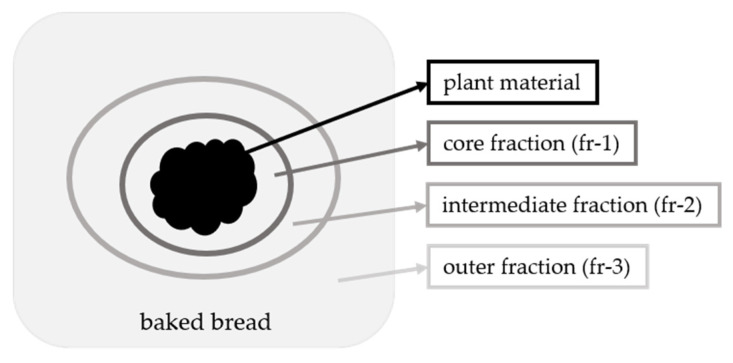
Scheme of the vegetable-enriched model bread application No III (bolus application). The crumb is separated into three fractions: core fraction (fr-1), intermediate fraction (fr-2), and outer fraction (fr-3).

For the bread with the freeze-dried nasturtium bolus in the center of the dough (application No. III), the plain dough was treated according to program section number 8. Then, the additional plant material was placed into the dough and the treatment was continued with program sections number 9 and number 10. The BG amount was around 17 µmol/100 g bread dough. After cooling down, the crust and the plant material were removed. The crumb was separated into three further fractions as shown in Figure 2: Crumb material in direct and close contact to the freeze-dried material was the core fraction (fr-1). With increased distance of the original bolus, an intermediate fraction (fr-2), and an outer fraction (fr-3) were prepared.

The separated garden cress material from application No. II and nasturtium from application No. III were also used for a GLS analysis to verify the loss of GLS and/or breakdown product formation resulting from the baking process or further transformations of the plant material. This further enables one to evaluate indirectly the leaching and migration into the dough. Additionally, an experiment was performed to investigate a possible heat-induced breakdown of GLS during the baking process of application No. III. For this, homogenized nasturtium was boiled in water for 10 min to deactivate the myrosinase and freeze-dried afterwards. The samples were then parted: one part was used for GLS analysis. The other part was baked into the bread, freeze-dried and also analyzed for the GLS content.

### 2.4. Protein Content

For the determination of the protein content the control breads were used and a traditional Kjeldahl protocol was performed. Freeze-dried bread (2.5 g) was mixed with 20 mL sulfuric acid (96–98%, p.a.) and a catalyst tablet. The reaction was heated for 4 to 5.5 h, till the solution was clear. For the distillation, a distillation unit (Büchi Labortechnik GmbH, Essen, Germany) was used. To the cooled down reaction mixture, 75 mL of water and 75 mL of aqueous sodium hydroxide (33%, *w*/*v*) were added, and steam distilled for 5 min at 75% steam pressure into 50 mL aqueous boric acid (4%). Finally, the distillates were titrated with 0.1 M hydrochloric acid until a color change from green to grey/colorless of the Tashiro indicator was observed. The calculation of the protein content was based on the nitrogen content and a protein conversion factor of 5.7 [27].

### 2.5. Enzymatic Hydrolysis of the Samples

For the extraction of ITC-amino acid conjugates, a combination of different enzymatic digestion protocols were used. First, the protocol of Pasini et al. was applied, in which an in vitro digestion was imitated to hydrolyze the proteins into peptides [28]. Therefore, 50 mg of freeze-dried bread was mixed with 4 mL of a pepsin solution (0.05 mg/mL in 0.2 M hydrochloric acid, enzyme/protein ratio: 1:30) and incubated for 30 min at 37 °C and 400 rpm in a thermoshaker (Hettich Benelux B.V., Geldermalsen, the Netherlands). Afterwards, 1.15 mL of a pancreatin solution (0.25 mg/mL in 1 M boric acid and 0.5 M sodium hydroxide solution, pH 6.8, enzyme/protein ratio 1:21) were added to the samples with a resulting pH value of 7.6. The reaction mixture was incubated again (37 °C, 400 rpm) for 150 min and the enzymatic digestion was finally stopped by the addition of 1 mL 20% (*w*/*v*) trichloroacetic acid (TCA). The reaction mixture was allowed to precipitate for 60 min, centrifuged for 10 min at 2576× *g*, and the supernatant was freeze-dried. The latter was diluted in 2 mL PBS-buffer and for further protein digestion, the protocol described by Kühn et al. was used with some modifications [23]. A pronase E solution (60 µL, 7 U/mg, 10 mg/mL in PBS buffer, enzyme/protein ratio 1:100) were added and the mixture was incubated for 18 h at 37 °C in a thermoshaker (400 rpm). The enzymatic digestion was stopped by adding 100 µL 20% (*w*/*v*) TCA and the extracts were centrifuged for 10 min at 24,104× *g*.

The supernatants were purified using solid phase extraction (SPE). For the SPE, a C18ec column was used, which was conditioned with methanol (3 mL) and equilibrated with formic acid (FA, 3 mL, 0.1% in water, *v*/*v*). Subsequently, the samples were applied and washed twice with aqueous FA (3 mL, 0.1% in water, *v*/*v*). The amino acid conjugates were eluted with methanolic FA (3 mL, 0.1% in methanol, *v*/*v*). The extracts were evaporated to dryness under a continuous stream of nitrogen and finally concentrated in 100 μL FA (0.1% in methanol/water, 80:20, *v*/*v*).

### 2.6. HPLC-ESI-MS/MS Analysis of Protein Conjugates

For LC-ESI-MS/MS analysis of the ITC protein conjugates, the protocol described by Kühn et al. was used with some modifications [20]. An aliquot (4 μL) was injected into the LC-ESI-MS/MS-system. For quantification, an external calibration with BITC-lysine (BITC-Lys) conjugate synthesized by Kühn et al. was used in a concentration range from 0.01 to 4 µmol/L [23]. The LC-ESI-MS/MS system consisted of a 5500 QTrap triple quadrupole MS/MS system (AB Sciex Germany GmbH, Darmstadt, Germany) combined with an Agilent 1260 Infinity II HPLC-system (Agilent Technologies Deutschland GmbH, Waldbronn, Germany). For data acquisition and analysis, the software Analyst 1.7.0 (AB Sciex Germany GmbH) was used.

The separation of the analytes was performed on a Kinetex^®^ C18 column (5 μm, 100 Å, 150 mm × 2.1 mm; Phenomenex Ltd., Aschaffenburg, Germany). The autosampler temperature was set to 4 °C and the column oven to 20 °C. The separation was performed with a flow rate of 300 μL/min of the mobile phase, consisting of 0.1% FA in water (eluent A) and 0.1% FA in MeOH (eluent B). At the beginning, the gradient consisted of 90% eluent A for the first minute. Subsequently, the concentration of eluent B increased linearly to 90% within 8 min and remained constant for 1 min. Afterwards, the start composition of eluent A and B was reached within 1 min. Finally, the column was re-equillibrated for 4 min with a composition of 90% eluent A.

The MS system was set to positive ionization mode with an entrance potential of 10 V. The ion spray voltage consisted of 5.5 kV, the desolvation gas temperature was 550 °C, the ion source gas pressure 70 psi for gas 1 and 55 psi for gas 2, and the curtain gas pressure was 40 psi.

### 2.7. HPLC-UV Analysis of Desulfo-GLS

The extraction of GLS and a conversion into desulfo-GLS were performed as previously described by Wiesner et al. with some modifications [29]. For extracting GLS from 10 mg freeze-dried plant material, 750 μL methanol (70% in water, *v*/*v*, 70 °C) were added as well as 25 μL of a *p*-hydroxybenzyl GLS solution (sinalbin, 1 mM in water) as internal standard. The mixture was heated at 70 °C for 10 min and afterwards centrifuged at 7748× *g* for 5 min at room temperature. The residue was re-extracted twice with 500 μL methanol (70% in water, *v*/*v*, 70 °C) and an incubation time of 5 min. All supernatants were combined.

To clean-up the extracts, small glass columns containing 500 μL of DEAE-Sephadex A-25 were used. The sorbent was activated with 6 M imidazole (2 mL, in FA 30% in water) and washed two times with water (1 mL), before sample-extracts were applied. Afterwards, 75 μL of purified β-glucuronidase (from *Helix pomatia*) were added and incubated overnight. Then, desulfo-GLS were eluted with 2 × 500 μL of water, transferred to Corning^®^ Costar^®^ Spin-X^®^ centrifuge tube filters (Merck KGaA) and centrifuged for 2 min at 5165× *g*. The sample solutions passed through the filters were used for HPLC-UV analysis.

An Agilent 1260 Infinity II LC system with an UV detector, equipped with a Poroshell 120 EC-C18 column (2.7 μm, 2.1 mm × 100 mm), was used for the separation. The autosampler temperature was set to 4 °C and the column oven to 23 °C. The mobile phase consisted of water (eluent A) and acetonitrile (eluent B) with a flow rate of 400 μL/min. In the beginning, eluent A was set to 99.8% for 2 min. Subsequently, the concentration of eluent B was linearly increased to 19.8% within 10 min and hold for 2 min. After that, the concentration of eluent B was further increased to 50% within 1 min and was kept constant for 1 min. Re-equilibration of the column was finally performed for 2 min with 99.8% of eluent A.

The identification of the desulfo-GLS based on a comparison of the retention time with reference GLS such as BG, 4-(methylsulfinyl)butyl glucosinolate and 2-propenyl glucosinolate at 229 nm. The quantification was done with *p*-hydroxybenzyl glucosinolate as internal standard and the specific response factor for each compound [30].

### 2.8. GC-MS Analysis of GLS Breakdown Products

Freshly frozen and ground bread (500 mg) was weighed frozen into a solvent resistant vessel. The extraction started with frozen bread powder that was allowed to heat to room temperature after adding the first round of methylene chloride. GLS breakdown products were extracted and analyzed with gas chromatography-mass spectrometry (GC–MS) using the extraction protocol described by Wermter et al. and the GC-MS protocol described by Hanschen et al., except that the transfer line temperature was set to 250 °C [31,32].

### 2.9. Statistical Analysis

For statistical analysis Statistica 64 (Version 13, Dell Inc., Tulsa, OK, USA) was used. The differences between the results of the two different applications No. I and No. II, and the non-processed and baked plant material was analyzed using the *t*-test. A statistical confidence level of 95% (*p* ≤ 0.05) was defined.

## 3. Results

### 3.1. Quantification of GLS in Nasturtium and Garden Cress before and after the Baking Process

In this experiment, the thermally-induced degradation of GLS during the baking process was investigated with a main focus on glucotropaeolin (BG) and its breakdown product BITC. For a comparison, the GLS content of the plant materials (*Tropaeolum majus* L., *Lepidium sativum* L.) was analyzed before and after the baking process. The results are shown in Table 2.

In the non-processed nasturtium (*Tropaeolum majus* L.), the GLS profile consisted of only BG with a content of 11.5 µmol/g freeze-dried material, which results around 0.5 µmol BG/g bread before the baking process for application No. I and 0.18 µmol BG/g bread for application No. III. In the separated material after the baking process of the nasturtium in application No. III, no BG could be detected, which corresponds to a release and possible degradation of 100% BG during the baking process.

**Figure 3:**
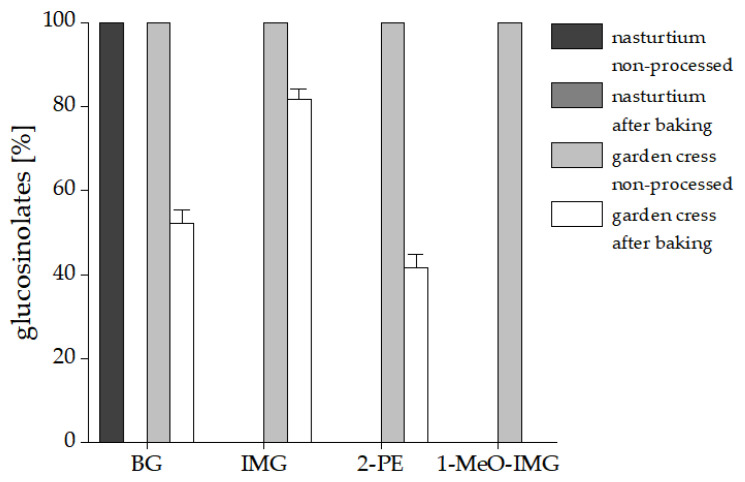
Thermal degradation of GLS in nasturtium and garden cress during the baking process. Results are expressed in % of residual glucosinolates of three treatment replicates analyzed in technical duplicates. Abbreviations: BG: benzyl glucosinolate, IMG: indolyl-3-methyl glucosinolate, 2-PE: 2-phenylethyl glucosinolate, 1-MeO-IMG: 1-methoxy-3-indolylmethyl glucosinolate.

The GLS profile of the fresh garden cress (*Lepidium sativum* L.) microgreens consisted mainly of BG with an amount of 48.80 µmol/g in freeze-dried material, which corresponds to an amount of 12.14 µmol/g in fresh material and around 0.2 µmol BG/g bread in application No. II. It also contained 0.36 µmol/g indol-3-ylmethyl glucosinolate (glucobrassicin, IMG), 0.45 µmol/g 2-phenylethyl glucosinolate (gluconasturtiin, 2-PE), and 0.08 µmol/g 1-methoxy-indol-3-ylmethyl glucosinolate (neoglucobrassicin, 1-MeO-IMG) in freeze-dried material. After the baking process, the BG content in garden cress microgreens decreased to 27.28 µmol/g freeze-dried material, which corresponds to a decline of 44.0%. The reduction of the other GLS in the garden cress differed from 17% to 100% in the following order: 1-MeO-IMG > 2-PE > BG > IMG (Figure 3).

With regard to the BG degradation and release of BITC, more BG was degraded when using the homogenized nasturtium leaves instead of the fresh garden cress microgreens, which could lead to a higher concentration of BITC in bread with freeze-dried material.

**Figure 4:**
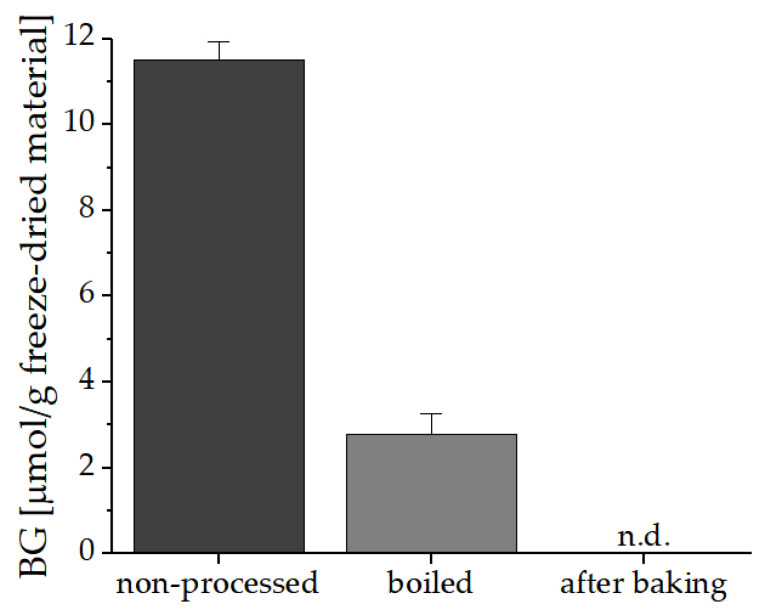
Thermal degradation of GLS in nasturtium after boiling in water for 10 min to deactivate the myrosinase and further baked into bread. The experiment and analysis were performed in duplicate. Abbreviations: BG: benzyl glucosinolates, n.d.: not detected.

For the additional experiment where the myrosinase was deactivated by boiling the freeze-dried nasturtium material for 10 min, Figure 4 shows that even by adding the homogenized material to boiling water for 10 min BG is degraded by 75% from 11.51 µmol/g to 2.79 µmol/g in freeze-dried material. After the baking process no BG could be detected. Therefore, GLS in freeze-dried homogenized material can be strongly degraded by heat.

### 3.2. ITC-Protein Conjugates in Bread with Different Cress Genera

To investigate possible reactions between BITC and wheat proteins, homogenized plant material of nasturtium (*Tropaeolum majus* L) was used for creating a large reaction surface and compared to the addition of garden cress (*Lepidium sativum* L.) microgreens, being comparatively more compact in structure.

**Figure 5:**
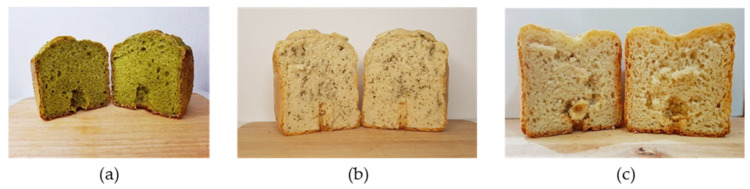
Pictures of the different cress-enriched model breads. (**a**) freeze-dried nasturtium (*Tropaeolum majus* L, 4%) mixed into the dough (application No. I); (**b**) fresh garden cress (*Lepidium sativum* L., 1.5%) mixed into the dough (application No. II); (**c**) reference bread without plant material.

The baked breads showed a difference in appearance, because of the differently applied plant materials. When adding a freeze-dried, powdered material, a homogenous green color of the crumb was obvious (Figure 5a), while the addition of fresh material was more heterogeneously distributed. So, individual green particles were noticed and spread all over the crumb (Figure 5b); the reference bread showed no further particularities (Figure 5c).

The LC-ESI-MS/MS method was developed for analyzing modifications with amino and thiol groups analyzed as ITC-lysine and ITC-cysteine conjugates. At hand of synthesized standards, the thiourea derivative BITC-lysine (BITC-Lys) and the dithiocarbamate BITC-cystein (BITC-Cys) can be quantified [20].

**Figure 6:**
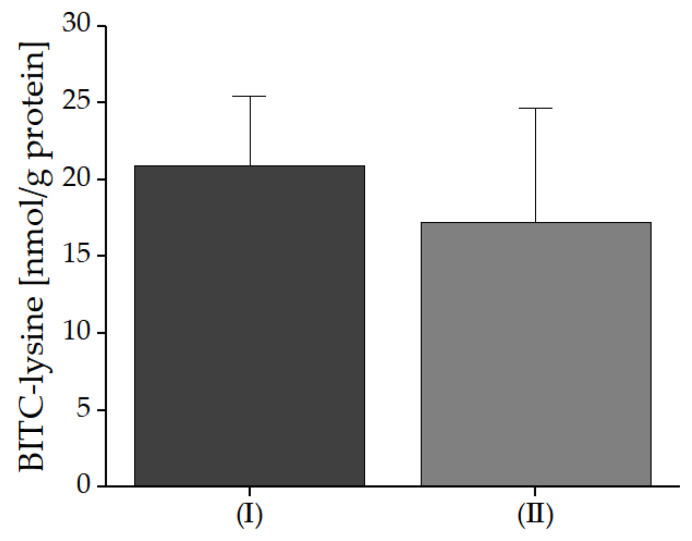
Formed BITC-Lys conjugates in bread with freeze-dried nasturtium (application No. I) and garden cress microgreens (application No. II) mixed into the dough. The breads were prepared and analyzed in triplicate. The initial amount of BG in the breads differs around 40%, but the amounts of BITC-Lys conjugates differed in a range of 10% per gram bread. Therefore, the addition of 4% homogenized nasturtium leaves resulted only in a 10% higher concentration of BITC-Lys conjugation compared to the addition of 1.5% fresh garden cress.

As shown in Figure 6, in the bread with freeze-dried powdered nasturtium leaves (*Tropaeolum majus* L., application No. I), 20.9 nmol BITC-Lys/g protein corresponding to 3.36 nmol BITC-Lys/g bread were determined, while in the bread with the fresh garden cress (*Lepidium sativum* L.) microgreens (application No. II), 17.2 nmol BITC-Lys/g protein (3.07 nmol BITC-Lys/g bread) were found.

In comparison to BITC-Lys, the dithiocarbamate BITC-Cys was not detected in the samples.

### 3.3. Quantification of BITC and Benzyl Cyanide in Bread with Different Cress Genera

Next to the identification and quantification of BITC-protein conjugates, the breakdown products of BG, BITC and the corresponding nitrile benzyl cyanide (BC), were investigated. This analysis was performed to obtain an indication of whether free BITC is detectable or whether the GLS has been transformed to the corresponding nitrile during the baking process. This would be visible in a low concentration of BITC and a high concentration of BC. Again, it was suspected that there might be a difference between the homogenized nasturtium leaves, and the fresh material of garden cress, due to the different degree of destruction of the plant material prior to and during the baking process.

**Figure 7:**
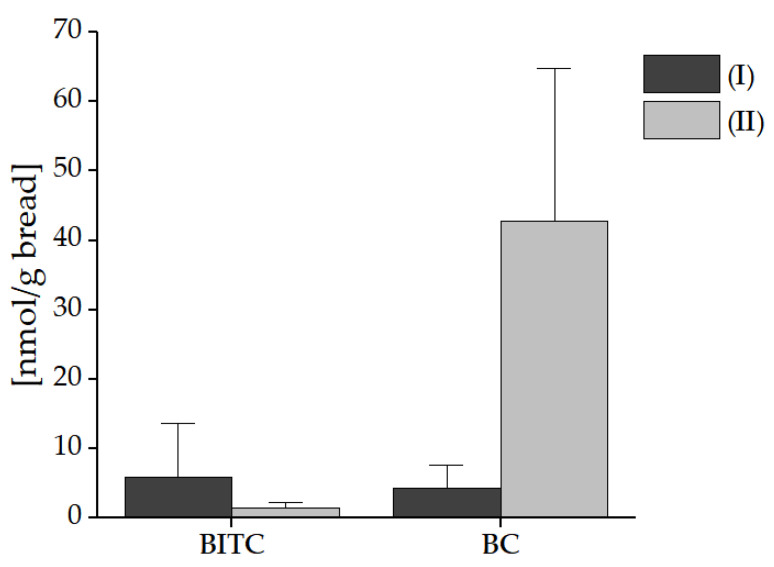
Formed benzyl glucosinolate breakdown products BITC and BC in bread with freeze-dried nasturtium (application No. I) and garden cress microgreens (application No. II) mixed into the dough. The breads were prepared in triplicate. Abbreviations: BITC: benzyl isothiocyanate, BC: benzyl cyanide.

The results in Figure 7 show only a slight difference (ratio of 1:1.38) in the concentration of BITC (5.93 nmol/g bread) and BC (4.30 nmol/g bread) for application No. I. In the bread application No. II, only 1.51 nmol BITC/g bread and 42.74 nmol BC/g bread were analyzed, resulting in a higher ratio of 1:28 BITC to BC. Comparing the different applications, more BITC was detected in application No. I, but 10 times more BC was analyzed in application No. II.

### 3.4. ITC-Protein Conjugates in Bread with a Centrally Placed Nasturtium Bolus

**Figure 8:**
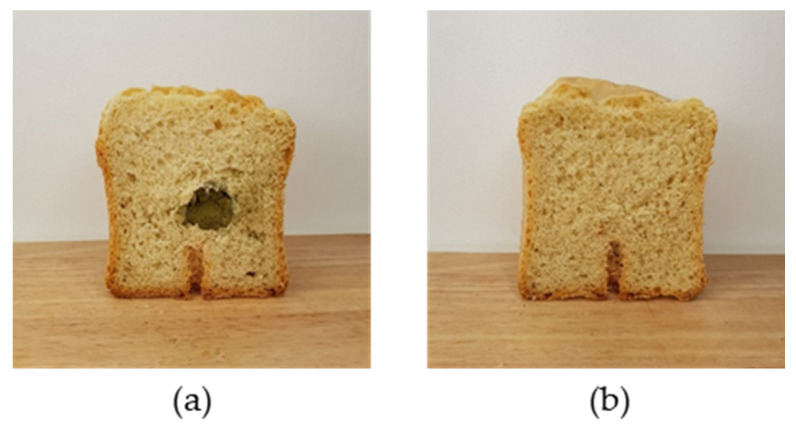
Picture of the cress-enriched model bread. (**a**) with freeze-dried nasturtium (*Tropaeolum majus* L, 1.5%) placed as centrally bolus (application No. III); (**b**) Picture of the reference bread without plant material.

The migration behavior of ITC into the food matrix and the distribution of reaction products during food processing were further studied in a model bread, where a bolus of freeze-dried nasturtium material was placed centrally (application No. III). Following the baking process, the bolus was removed and the GLS content analyzed. The bread was divided into a core, an intermediate, and an outer fraction (Figure 2). The appearance of the crumb did not show any differences compared to the reference bread (Figure 8a,b).

**Figure 9:**
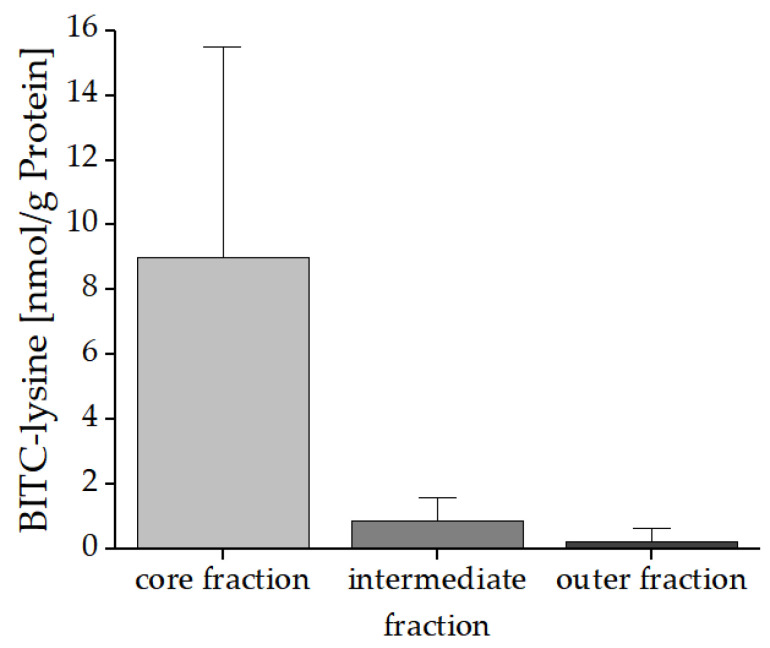
Amounts of the BITC-Lys conjugates in the core, intermediate, and outer fraction of the bread with freeze-dried nasturtium added selectively (application No. III). Three different breads were prepared and analyzed in triplicate.

BITC-Lys was detectable in the crumb. As anticipated, the highest concentration with 9.02 nmol BITC-Lys/g protein (1.38 nmol BITC-Lys/g bread) was found in the core fraction, while the analysis of the intermediate fraction showed a lower concentration of only 0.89 nmol BITC-Lys/g protein (0.12 nmol BITC-Lys/g bread) (Figure 9). In the outer fraction BITC-Lys could still be detected with a concentration of 0.26 nmol BITC-Lys/g protein (0.02 nmol BITC-Lys/g bread). Again, the BITC-Cys conjugate could not be detected in any of the fractions.

### 3.5. Quantification of BITC and Benzyl Cyanide in Bread with a Centrally Placed Nasturtium Bolus

**Figure 10:**
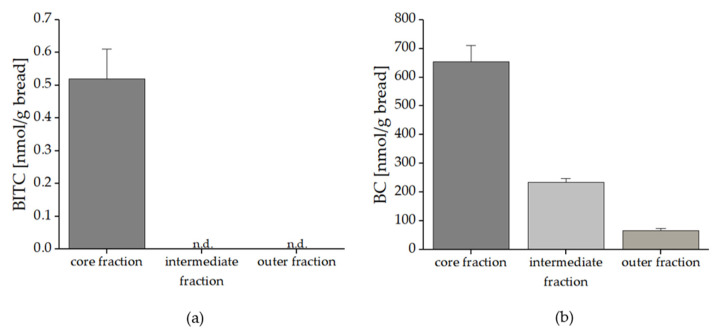
Formed benzyl glucosinolate breakdown products BITC (**a**) and BC (**b**) in bread with freeze-dried nasturtium added as a bolus in the middle of the bread (application No. III). The breads were prepared in triplicate. Abbreviations: BITC: benzyl isothiocyanate, BC: benzyl cyanide.

It was also of interest to investigate the migration behavior of the BG breakdown products BITC and BC in the different fractions of the bread. As shown in Figure 10a, it was found that only in the core fraction BITC could be detected with a concentration of 0.52 nmol/g bread. BC was found in all three fractions with decreasing concentration from the core fraction to the outer fraction. In detail the results in Figure 10b show that in the core fraction 653.02 nmol BC/g bread was found, decreased by 64% to the intermediate fraction (235.25 nmol BC/g bread). The difference from the intermediate to the outer fraction (66.98 nmol BC/g bread) was around 70%.

## 4. Discussion

In the present study, the degradation of GLS, the migration of their main breakdown product ITC, the corresponding nitrile, and the formation of ITC-protein conjugates were exemplarily investigated in a vegetable-enriched bread.

For these studies, bread was baked with parts of fresh garden cress (*Lepidium sativum* L.) microgreens and homogenized freeze-dried powdered nasturtium leaves (*Tropaeolum majus* L.). The different cress genera mainly contained the GLS BG, which can be degraded to BITC and the corresponding nitrile. This allowed a direct conclusion on the used material (homogenized or microgreens), independent of different chemical structures and therefore, different degradation rates of GLS during a heat treatment [33]. Because of the different percentage of addition the bread with nasturtium contained 2.5 times more BG before the baking process, therefore it was assumed that there is a more intense release of BITC during the baking process and consequently to an enhanced conjugation with proteins.

With regard to the GLS profile before and after the baking process, 100% BG in the homogenized freeze-dried nasturtium were degraded. In comparison, only 44% BG were released during the process from the garden cress microgreens into the crumb. Therefore, in application No. I around 50 µmol/100 g bread dough were degraded and in application No. II around 9 µmol BG/100 g bread dough. A former study assumed that due to a low destruction of the plant matrix, the GLS remain longer intact in the plant material [24]. The results of the present study underlined this assumption. When using fresh, non-homogenously material, the migration of released BITC into the surrounding food matrix might be hampered by the intact plant structure and the reduced reaction surface in comparison to a freeze-dried material (Figure 5b). The plant tissue of freeze-dried material is already more or less disintegrated. By adding the material to the moist bread dough, the GLS could already be degraded by the enzyme myrosinase before the baking process, which is a difference to the use of garden cress microgreens. During the baking process, further heat-induced degradation can occur. The more homogeneous, pulverized material also leads to easier migration of the GLS and their breakdown products into the surrounding matrix before and during the baking process because the homogenized material ensures a broader distribution in the crumb (Figure 5a).

For these reasons and the higher BG concentration it was assumed that there would be significantly more protein conjugates in application No. I. The results confirmed that there is indeed a higher concentration of BITC-Lys, but it is only 10% higher than in application No. II even though the BITC levels likely formed were lower, because only 44% BG is released. To explain these results, a correlation can be made to the analyzed breakdown products. The breakdown product results show that there is only a slight difference in the amount of unreacted BITC in the different breads. However, the BC results show a 10-times higher concentration in the bread with fresh garden cress compared to the bread with freeze-dried, homogenized nasturtium. Thus, overall, a higher amount of breakdown products is detectable in application No. II.

The difference can be explained by the different states of the plant materials. In application No. I, BG might be already enzymatically degraded to BITC before the baking process began. This BITC might be degraded by heat action at an early stage of the baking process and thus might be no longer available for follow-up reactions. In addition, there appears to be volatilization of BITC and BC, so that overall fewer breakdown products were detectable in the baked bread of application No. I. When fresh material is used, the degradation of BG starts probably mainly during the baking process, so that initially the released BITC is available for reactions with proteins directly during the baking process. BC very likely is formed in high amounts due to thermal degradation of BG, as nitriles have been found to be the main degradation products from GLS in heated vegetables [13,25]. Therefore, despite the lower initial concentration of BG in application No. II, a larger amount of BITC may have been available for the formation of protein conjugates, as shown in the results of the ITC conjugates.

In the present study, 0.01% of BG in application No. I and 0.02% in application No. II were transformed into BITC-Lys conjugates, resulting in around 0.01% modified lysine in both breads. In an experiment described by Kühn et al., curd was mixed with cress, resulting in a typical German spread (‘Kräuterquark’) and analyzed for possible ITC protein conjugates. The results showed a higher conjugation of BG to BITC-Lys of up to 2–4% [23]. Considering the pH value, which not only influences the formation of ITC conjugates, but also their stability, more lysine conjugates should be detectable in wheat bread (pH 5.9) than in curd (pH 4.5), since a pH value close to the neutral range leads to an increased formation and stabilization of thioureas as shown by Platz et al. [34]. Therefore, the different results could be explained by the far different processing approaches. Baking produces temperatures of up to 95 °C for around 30 min in the bread matrix (Figure 1), whereas curd with herbs is only mixed at room temperature.

In regard to the enzymatic degradation of GLS selected studies showed that a short heat exposure of white cabbage (*Brassica oleracea var. capitata f. alba* cv. Minicole), red cabbage (*Brassica oleracea var. capitata f. rubra* cv. Primero), and kohlrabi (*Brassica oleracea var. gongylodes cv.* Kolibri,) for 10 min at around 60 °C led to an increased ITC formation due to inactivation of specifier proteins that enhance enzymatically nitrile and epithionitrile formation. In contrast, longer heat exposure durations (of up to 120 min), especially in the range of 100 °C, led to a nitrile formation due to thermal GLS degradation. Nitriles then accumulate in the matrix, because of their heat stability. Compared to the nitriles, the concentration of ITC decreases with prolonged heating, which results in less ITC available for follow-up reactions with proteins [13]. During the baking process, ITC could decompose to a wide range of further breakdown products, depending on their chemical structure. As an example for an aromatic ITC, a study applying phenethyl isothiocyanate (PEITC) in aqueous solutions showed that PEITC can be thermally decomposed to phenethylamine and can further react to *N,N′*-diphenethylthiourea [35]. This hydrolysis reaction of ITC results in less protein conjugates in heated food products because less ITC is available for follow-up reactions with proteins.

To understand not only the formation of ITC-protein conjugates, but also the migration of GLS, ITC, and possible reaction products, another bread with a centrally placed freeze-dried nasturtium bolus was prepared. This experiment showed that BITC migrated up to the outer fraction and the concentration of BITC must have been still high enough to detect BITC-protein conjugates. It could be demonstrated that the concentration of the conjugates was reduced by 90% from the core fraction to the intermediate fraction (Figure 7). However, ITC-protein conjugates are still detectable in the outer fraction reduced by 97% to the core fraction.

The analysis of the BG breakdown products BITC and BC showed, that BITC was only detectable in the core fraction, whereas BC, which was highly abundant, could be quantified in all three fractions reduced by 90% from the core fraction to the outer fraction. This result shows that BITC migrates to the outer fraction during the baking process and reacts with proteins. Furthermore, not only the BITC migrates from the core fraction to the outer fraction, as proven by the protein adducts, but also formed BC.

In comparison to application No. I with homogenized nasturtium leaves mixed into the dough, there might also be a difference of degradation of BG. Whereas BG might be mainly degraded enzymatically in application No. I due to the contact with the dough, the BG in freeze-dried material added as a bolus might be degraded as well by the heat during the baking process, because not all the material is in contact with the moisture of the bread dough. When adding homogenized nasturtium to boiling water and treating it for 10 min at 100 °C, already 75% of BG were degraded. Therefore, BG seems to be very heat labile, and this might explain the total degradation of BG that was observed.

In this study, 1.5% to 4% homogenized nasturtium was applied to the bread doughs. In a former study, bread has been enriched with vegetable powder in a range of 1–3%, which lies within the range of quantity used [36]. Studies using fresh diced vegetable ingredients, such as kale, beetroot, and spinach, enriched the breads in a range of 10 to 40% [6,24,37]. Here, the prepared breads with fresh garden cress microgreens were enriched with 1.5%. With regard to a higher enrichment around 10 to 40% the concentration of BG and thus, resulting BITC likely would further increase significantly. This again can lead to a more intense release and migration of ITC and more follow-up reactions with proteins.

In all experiments, only BITC-Lys conjugates were detected, although the calculated difference in the concentrations of lysine (2.3% in wheat proteins) and cysteine (1.6% in wheat proteins) in the initial wheat proteins was only 0.7%. In another study, where garden cress was implemented in curd, higher concentrations of BITC-Cys conjugates were detected, despite the lower concentration of cysteine (0.3%) to lysine (8.2%) of the milk proteins [23]. Because of these results it was assumed that more cysteine conjugates would be formed in the present study, which could not be confirmed. These differences can be explained by the pH value and the heat treatment during the baking process (Figure 1). The pH value of the food matrix not only influences the formation of BITC protein conjugates but also their stability. The pH of the bread (5.9) should lead to a higher formation of BITC-Lys conjugates [34]. Additionally, BITC-Cys conjugation is reversible and shows only little stability with increasing temperature, whereas reaction between the amino group of lysine and ITC are stable and temperature has only a minor influence on BITC-Lys conjugates [20,38,39].

Next to a potentially lower biological value, protein conjugations have further consequences. In former studies, an influence on typical digestive enzymes has already been observed: While trypsin and chymotrypsin were not able to fully hydrolyze ITC-modified proteins, pepsin activity was not hindered. These differences are assigned to the typical specificities of those enzymes to split near selected amino acids. Trypsin, for example, hydrolyzes peptide linkages containing lysine or arginine, whereas pepsin is a non-specific protease [19,40,41,42]. Therefore, the digestibility by trypsin can be more affected due to the BITC-Lys conjugation. Next to the influence on the digestibility, another experiment showed that ITC modifications also lead to a decrease of enzyme activity as shown with tyrosine phosphatase [43]. In food production, a reduced water solubility of proteins due to a conjugation with the hydrophobic BITC could be relevant, as well as, altered heat aggregation, foaming and emulsifying properties which was demonstrated at hand of AITC modified β-lactoglobulin [41,44].

In addition to GLS, Brassicales vegetables also contain other SPM such as flavonoids and carotenoids. The heat exposure during the baking might also affect these SPM, which could also lead to follow-up reactions [24,45]. It has already been shown that the antioxidant activity of phenolic compounds in vegetables is often not diminished, even when there is a thermally-induced breakdown to smaller compounds, but there is also a formation of complexes with proteins during the baking process [45].

## 5. Conclusions

In foods containing proteins and GLS-rich vegetables, reactions between ITC and proteins may occur and the reaction of BITC with lysine can lead to a lower biological value of the protein [46]. However, in comparison to non-heated foods, less protein conjugates are formed in heated food products, because the formation of ITC during the baking process is diminished due to evaporation and hydrolysis reactions. Therefore, a lower bioavailability of lysine due to reactions with ITC is less of a concern in heated processed foods, but there is also no health benefit from more available ITC.

In addition to the ITC-amino acid conjugates with cysteine and lysine investigated herein, there could be further reactions with other amino acids, such as methionine (1.1% in the bread) and tyrosine (1.5% in the bread). Such reactions have not yet been investigated and could lead to a higher ITC-protein conjugation than previously anticipated [47,48]. It could also be interesting to study how the concentration of conjugates change over a longer period of time, because bread is typically consumed over several days.

## Figures and Tables

**Table 1:** Baking program of the UNOLD Backmeister^®^ extra Modell 65811.

Section Number	Section Name	Time [min]	Temperatures Ø [°C]
1	tempering	17	22.4
2	slow stirring	3	25.6
3	kneading clockwise	2	25.6
4	kneading anti-clockwise/clockwise	13	27.8
5	1st rising without heat	45	28.0
6	smoothing dough	1	28.0
7	2nd rising without heat	18	26.8
8	smoothing dough	1	26.8
9	3rd rising with heat	45	33.6
10	baking	55	87.8
	total	200	

**Table 2:** GLS profiles and contents in nasturtium (*Tropaeolum majus* L.) and garden cress (*Lepidium sativum* L.) material, either non-processed (before baking) or after the baking process. Results are presented in µmol/g dry weight (DW) ± standard deviation. Because of the use of fresh garden cress the result of benzyl glucosinolate is additionally expressed in fresh weight (FW) for this material. Abbreviations: n.d.: not detected, BG: benzyl glucosinolate, IMG: indol-3-ylmethyl glucosinolate, 2-PE: 2-phenylethyl glucosinolate, 1-MeO-IMG: 1-methoxy-indol-3-ylmethyl glucosinolate.

		Nasturtium		Garden Cress	
		Non-Processed	After Baking ^1^	Non-Processed	After Baking ^1^
BG	DW	11.51 ± 0.42	n.d.	48.80 ± 3.78	27.28 ± 2.92
	FW	-	-	12.14 ± 0.94	6.79 ± 0.73
IMG	DW	-	-	0.36 ± 0.15-	0.54 ±0.06-
2-PE	DW	-	-	0.44 ± 0.07	0.29 ± 0.13
1-MeO-IMG	DW	-	-	0.08 ± 0.01	n.d.

^1^ Results of three treatment replicates analyzed in technical duplicates.

## Data Availability

The data sets presented in this study are available on request from the corresponding author.
